# The “new” normal

**DOI:** 10.3205/zma001444

**Published:** 2021-02-15

**Authors:** Sigrid Harendza

**Affiliations:** 1 Universitätsklinikum Hamburg-Eppendorf, III. Medizinische Klinik, Hamburg, Germany

## Editorial

We find so many things normal in everyday life. That one can press a light switch, that one shakes hands with people to greet them, or that one wears a seat belt in the car. Some of us will remember the days well when it was far from "normal" to wear a seat belt in a car. And yet it feels “normal” meanwhile. In medicine, too, there are such developments towards a “new” normality that does not feel so “new” anymore after a while. The fact that dentists wear masks and gloves and sometimes even protective goggles [1], although there is now a hepatitis B vaccination, seems quite normal to me, although this was not the case in my childhood. The fact that I wear gloves to draw blood in order to protect myself from infections [2] is something I only learned during my internship year. At first it seemed quite tiring to me. Today I find it completely normal. And now SARS-CoV-2, which makes me, an internist, wear a surgical mask every day. This may become a “new” normal in hospitals, as it has since been discovered that general surgical mask wearing also protects against transmission of seasonal cold and flu [3]. But will I learn to think of this as “normal”?

How changes or innovations spread and are adopted at the individual level is described by sociologist Everett M. Rogers in his diffusion theory [4]. In the process of adopting a change, the early adopters are followed by the early majority, then the late majority, and finally the laggards. Whether a change has “really” arrived, i.e., reached a status quo, can be determined by the language used by the adopters. It is therefore easy to guess what it means for the acceptance of a change if there is continued talk of a “new” curriculum, “new” licensing regulations, “new” federal states or a “new” normal. Adopting some changes takes time, sometimes decades. The COVID-19 pandemic will not give us that much time to establish a “new” normal for the study of medicine, dentistry, veterinary medicine, and other health professions.

The first steps in the direction of a “new” normal have definitely already been taken with the digitalization of courses. Who would have thought a year ago that digital lectures would work so well, be well received by students, be rated very highly, and be attended much more frequently than face-to-face lectures [5]. Moreover, there are even already instructions on how to integrate electronic courses into one’s own teaching [6]. Why should a course in histology or radiology not be taught digitally on a permanent basis, if the corresponding microscopic or radiological images can be seen much better on the home computer screen than on the screen in the seminar room? Hoping that the SARS-CoV-2 vaccinations will result in courses being taught as “normally” as before is probably not an appropriate strategy. Moreover, for some courses, returning to the “old” normal is not even reasonable. But running the courses digitally only for an indefinite period of time, even though good concepts have been established for this [7], is probably not a sustainable solution either to acquire medical skills and attitudes. Therefore, it will be necessary to think out of the box in the longer term [8].

The distance regulations and the wearing of a mouth-nose protection will not disappear from our everyday life for the time being. Some known teaching concepts can be established in a way that fulfills these regulations [9]. However, completely new concepts will also be necessary. Perhaps it would be good to complete ten instead of four clinical clerkships, because individual students are much easier to integrate into the daily work on hospital wards and in practices under the prevailing hygienic conditions than small groups of students during bedside teaching or block placements? Presumably, we will need new communication courses to practice communicating with masks and learn other mimic response patterns. Or perhaps a semester should run from April 1^st^ to September 30^th^ because SARS-CoV-2 infections are less likely to occur during those months, and the rest of the year would be lecture-free time? Maybe we need the personality trait conscientiousness as a new criterion for student selection, so that we can better trust that students and physicians adhere to regulations and less danger to patients occurs? Much more is conceivable and may be thought of and should be put to the test.

Whatever the “new” normal will look like at universities and in medical and health professions programs: inappropriate treatment of students in interships, as reported by Bormuth et al. in this issue [10], should not happen anymore. Medical care of asylum seekers will continue to be an important topic for medical students in the “new” normal. The development of content for such a multidisciplinary course as a clinical elective and the evaluation after its implementation are explained by Ziegler et al. [11]. Schlegel et al. demonstrate that learning content could move completely into the virtual space and that this can meet with intergenerational acceptance [12]. Additionally, self-directed learning in clinical clerkships, reported by Röcker et al. [13], will also be important in the future. The selection of teaching practices for the internship year will also play an important role in the “new” normal. Demmer et al. present a concept for this [14].

These and other papers in this issue of the GMS Journal for Medical Education demonstrate the importance of not dwelling on the hope of a return to the “old” normal of studying with the help of the SARS-CoV-2 vaccine, but proactively shaping the “new” normal of studying and everyday medical practice for and with our students in a responsible manner. In this way, studying and providing medical care to those entrusted to us can once again become the educational and professional normal.

## Competing interests

The author declares that she has no competing interests.
